# Editorial: Innovations in psychological care for oncology and palliative settings: a holistic approach

**DOI:** 10.3389/fpsyg.2026.1866283

**Published:** 2026-05-15

**Authors:** María Cantero-García, María Rueda-Extremera, Simon Dune

**Affiliations:** 1 Universidad a Distancia de Madrid (UDIMA), Madrid, Spain; 2 Dublin City University, Dublin, Ireland

**Keywords:** cancer survivorship, caregiver stress, cognitive-behavioral therapy, emotional adjustment, end-of-life care decisions

The present Research Topic is positioned at the intersection of a critical transformation in contemporary healthcare: the growing recognition that psychological care is not a complementary dimension of oncology and palliative medicine, but a fundamental determinant of patient outcomes, quality of life, and the ethical integrity of care itself. As the global burden of cancer and life-limiting illness continues to rise, so too does the urgency of addressing the complex psychological, relational, and existential dimensions that accompany these conditions. Illness, in this context, cannot be reduced to a biological disruption; it is a deeply human experience that reshapes identity, challenges meaning, and reconfigures relationships. The contributions gathered in this Research Topic respond to this paradigm shift by advancing a holistic, integrative, and context-sensitive understanding of psychological care—one that moves beyond symptom reduction to embrace the full complexity of human adaptation to illness. Across diverse methodologies, cultural settings, and clinical populations, these studies collectively redefine the scope of psycho-oncology and palliative psychology, positioning them as central to the development of more humane, responsive, and effective healthcare systems.

A first major thematic axis emerging from this collection concerns the role of structural, socioeconomic, and systemic determinants in shaping psychological experiences of illness. These contributions converge on a critical insight: psychological distress in oncology is not solely an individual phenomenon, but is deeply embedded in material conditions, healthcare infrastructures, and access to resources. In this regard, Belay et al. provide a compelling qualitative analysis of psychosocial service provision across six hospitals in Ethiopia, identifying structural barriers such as workforce shortages, limited financial resources, and the prioritization of somatic care over psychosocial support. At the same time, they highlight contextually grounded facilitators—including survivor narratives and structured supervision—that offer pathways for improving care even in resource-constrained environments.

Extending this structural perspective, Zou et al. conceptualize financial toxicity in liver cancer as a multidimensional construct that encompasses economic burden, psychological distress, and symptom experience, while emphasizing the buffering role of family resilience. Similarly, Osowiecka et al. document high levels of unmet supportive care needs among Polish cancer patients, particularly in informational and financial domains, revealing systemic gaps that directly affect wellbeing. Complementarily, Hu et al. demonstrate that social alienation is shaped by illness perception, fear of progression, and deficits in social support, reinforcing the importance of relational contexts. This broader ecological perspective is further enriched by Mok et al., whose cross-cultural analysis underscores how communication barriers and cultural expectations influence cancer care experiences among Chinese patients in the United Kingdom. Together, these studies establish that meaningful innovation in psychological care must address not only individual symptoms, but also the structural and cultural conditions that shape vulnerability and access to support.

Building upon this contextual foundation, a second thematic axis focuses on cognitive and emotional processes as core mechanisms of psychological adjustment. Across multiple contributions, constructs such as illness perception, fear of progression, rumination, and emotional regulation emerge as central determinants of patient outcomes. For instance, Xu et al. demonstrate that maladaptive illness perceptions significantly impair quality of life in lung cancer patients, both directly and indirectly through heightened fear of progression. Similarly, Wang et al. identify a mediated pathway in which hope and coping styles connect illness perception to self-transcendence in gastric cancer patients, highlighting the role of meaning-making processes in adaptation.

The importance of repetitive negative thinking is further evidenced by Huang et al., who show that rumination mediates the relationship between fear of progression and sleep disturbance in melanoma patients, suggesting that targeting cognitive processes may yield more effective interventions than symptom-focused approaches alone. In a configurational analysis, Gong et al.reveal that post-traumatic stress symptoms in breast cancer survivors arise from complex interactions between body image disturbance and fear of progression, illustrating the multifactorial nature of psychological distress. Complementing these findings, Zimmaro et al. introduce the construct of body compassion, demonstrating its specific role in reducing distress beyond general mindfulness. Likewise, García-Torres et al. establish psychological flexibility as a robust predictor of emotional wellbeing, fatigue, insomnia, and post-traumatic growth, reinforcing the relevance of acceptance-based therapeutic approaches. In contrast, Holst et al. highlight the limited predictive capacity of sociodemographic variables in explaining avoidant coping, pointing to the complexity and individuality of psychological responses to illness. Collectively, these studies underscore the need for process-oriented, theoretically grounded interventions targeting cognitive and emotional mechanisms.

A third thematic axis addresses resilience, coping, and adaptive processes across individual and relational systems. Moving beyond deficit-based perspectives, these contributions conceptualize adaptation as a dynamic and potentially transformative process. Duan et al. identify distinct profiles of post-traumatic growth among spouses of breast cancer patients, demonstrating that adaptation extends beyond the individual to encompass relational systems. Similarly, Li et al. show that dyadic coping plays a central role in shaping long-term wellbeing in patients with malignant bone tumors, highlighting the interdependence of emotional adjustment within intimate relationships.

At the individual level, Gao et al.provide evidence for the effectiveness of resourcefulness training in reducing cancer-related fatigue and fear of recurrence, while enhancing adaptive coping capacities. Complementarily, Wang et al.demonstrate that family resilience mediates the relationship between discharge readiness and self-management efficacy, underscoring the importance of family systems in recovery processes. Additionally, Liao et al.reveal that alexithymia negatively impacts quality of life through reduced social support and self-compassion, pointing to emotional awareness and self-relational processes as key intervention targets. In line with this integrative perspective, Weng et al.further demonstrate that patient-centered nursing and psychosocial care strategies significantly improve quality of life, emotional wellbeing, and functional outcomes in cancer patients, emphasizing the importance of addressing psychological, social, and physical dimensions simultaneously. These findings advocate for a multidimensional understanding of resilience that integrates individual, relational, and contextual resources.

A fourth thematic axis explores quality of life, psychological distress, and clinical outcomes across the continuum of care, challenging the assumption that favorable medical prognosis necessarily translates into psychological wellbeing. Kuang et al.demonstrate that patients with differentiated thyroid cancer experience persistent psychological distress despite positive clinical outcomes, highlighting the importance of monitoring patient-reported outcomes throughout the care trajectory. Similarly, Liu et al.identify significant psychological impacts associated with thyroid cancer diagnosis and treatment, shaped by socioeconomic and familial factors. From an ethical and patient-centered perspective, Mínguez-Garrido et al. highlight critical limitations in pediatric assent processes in oncology clinical trials, showing that although technical accuracy is generally adequate, information often fails to be accessible or meaningful for children. Their findings underscore the need for age-adapted communication strategies and the inclusion of young patient advisory groups to ensure genuinely informed and participatory decision-making.

The interaction between systemic crises and psychological vulnerability is illustrated by Luo et al., who report elevated levels of anxiety and depression among cancer patients during the COVID-19 pandemic. Meanwhile, Wang et al.identify key risk factors for depression in cervical cancer patients, including socioeconomic disparities and disease severity. From a biopsychosocial perspective, Ning et al. introduce genetic markers associated with trajectories of psychological distress, while Chen et al. explore the relationship between psychopharmacological treatment and immune function. These contributions collectively reinforce the need for integrative models that account for the interaction between biological, psychological, and contextual factors.

A fifth thematic axis encompasses innovations in psychological and psychosocial interventions, reflecting a significant expansion of therapeutic approaches and care models. Within evidence-based practice, Sancho-Martínez et al.provide a systematic review of fear of cancer recurrence, highlighting the effectiveness of cognitive-behavioral and third-wave interventions. Complementarily, Svozilová et al. propose a comprehensive synthesis of evidence on creative arts therapies, while D'Oria et al. demonstrate their impact on both physiological and psychosocial outcomes.

Importantly, this volume also incorporates innovative and non-traditional approaches. Carnero-Sierra conceptualizes therapeutic clowning as an affective and relational intervention capable of transforming clinical environments, while Perasso et al.advocate for play-based approaches as integrative frameworks in pediatric oncology. Additionally, Chen et al. and Liu and Guo demonstrate the effectiveness of structured educational and personalized nursing interventions, and Hua et al.further demonstrate that structured training in oncology psychology significantly enhances caring competencies among healthcare professionals, including empathy, communication skills, and patient-centered clinical decision-making. Their findings, based on a quasi-experimental design, highlight how integrating psycho-oncological education into medical training can improve both professional preparedness and the quality of patient care. In this context, Zhou et al. provide robust evidence supporting complementary and non-pharmacological approaches, demonstrating through a meta-analysis of randomized controlled trials that acupuncture-related therapies significantly reduce depression, anxiety, and sleep disturbances while improving overall quality of life in cancer patients. These findings highlight the growing relevance of integrative care models that combine biomedical and traditional therapeutic strategies. Together, these contributions illustrate that innovation in psychological care extends beyond technique to encompass new ways of conceptualizing care relationships, environments, and therapeutic processes.

A sixth thematic axis examines digital health and technology-mediated interventions, which are becoming increasingly relevant in contemporary healthcare systems. Kellermann et al. identify a critical gap in digital support during periods of diagnostic uncertainty, conceptualizing the “waiting room” as a psychologically vulnerable phase requiring targeted interventions. Complementarily, Varsi et al. demonstrate how digital stress-management interventions enhance self-efficacy, engagement, and emotional regulation through personalization and accessibility. These findings suggest that digital tools, when grounded in psychological theory, can significantly expand the reach and effectiveness of psychosocial care.

A seventh axis focuses on care transitions and self-management, highlighting the challenges patients face beyond the hospital setting. Letrecher et al. conceptualize the return home as a complex psychosocial transition involving identity reconstruction and the redefinition of everyday life, while Tan et al. reveal the difficulties patients encounter in translating clinical knowledge into real-world self-management practices. These studies emphasize the importance of continuity of care and the need to support patients in navigating life beyond clinical environments.

An eighth axis addresses the community and relational dimensions of care, emphasizing that psychological support extends beyond formal healthcare systems. Horicks et al. demonstrate the value of peer-support communities in reducing isolation and fostering empowerment, while Yang et al.identify key barriers and facilitators to help-seeking behavior, including beliefs, prior experiences, and family influence. These contributions highlight that care is co-constructed within social networks and that community-based resources play a crucial role in psychological adaptation.

A ninth and final thematic axis centers on healthcare professionals and the ethical dimensions of care. Xin et al. reveal the relationship between attitudes toward death and moral distress among oncology nurses, underscoring the emotional burden associated with end-of-life care. These findings are complemented by Belay et al. and Hua et al., which emphasize the importance of training, supervision, and institutional support in sustaining professional wellbeing and ensuring high-quality care delivery.

Taken together, the contributions in this Research Topic articulate a coherent and forward-looking vision for the future of psychological care in oncology and palliative settings. By integrating structural, psychological, relational, and technological perspectives, they demonstrate that innovation in healthcare cannot be reduced to technical advancement alone. Rather, it emerges from the capacity to understand illness as a lived, relational, and meaning-laden experience, and to design interventions that are responsive to this complexity.

In conclusion, this Research Topic offers more than a synthesis of current evidence; it advances a transformative agenda for rethinking psychological care in oncology and palliative contexts. The collective work presented here calls for a decisive shift toward models of care that are not only scientifically rigorous, but also ethically grounded, culturally sensitive, and deeply human-centered. It challenges the field to move beyond fragmented approaches and toward integrative frameworks capable of addressing the full spectrum of patient experience—from biological vulnerability to existential meaning. Ultimately, the contributions in this volume reaffirm a fundamental principle: that the true measure of innovation in healthcare lies not only in extending life, but in enhancing the quality, dignity, and meaning of that life, even—and especially—in the face of illness and finitude.

